# Hypoxia-mimetic agents inhibit proliferation and alter the morphology of human umbilical cord-derived mesenchymal stem cells

**DOI:** 10.1186/1471-2121-12-32

**Published:** 2011-08-09

**Authors:** Hui-Lan Zeng, Qi Zhong, Yong-Liang Qin, Qian-Qian Bu, Xin-Ai Han, Hai-Tao Jia, Hong-Wei Liu

**Affiliations:** 1 Department of Hematology, the First Affiliated Hospital of Jinan University, Guangzhou 510630, China; 2 Department of Hematology, Guangdong No.2 Provincial People's Hospital, Guangzhou 510317, China; 3 Department of Internal Medicine, the Third Affiliated Hospital of Nanfang Medicine University, Guangzhou 510630, China; 4 Institute of Life and Health Engineering, Jinan University, Guangzhou 510630, China; 5 Department of Plastic Surgery and Cosmetology, the First Affiliated Hospital, Jinan University, Guangzhou, 510630, China

## Abstract

**Background:**

The therapeutic efficacy of human mesenchymal stem cells (hMSCs) for the treatment of hypoxic-ischemic diseases is closely related to level of hypoxia in the damaged tissues. To elucidate the potential therapeutic applications and limitations of hMSCs derived from human umbilical cords, the effects of hypoxia on the morphology and proliferation of hMSCs were analyzed.

**Results:**

After treatment with DFO and CoCl_2_, hMSCs were elongated, and adjacent cells were no longer in close contact. In addition, vacuole-like structures were observed within the cytoplasm; the rough endoplasmic reticulum expanded, and expanded ridges were observed in mitochondria. In addition, DFO and CoCl_2 _treatments for 48 h significantly inhibited hMSCs proliferation in a concentration-dependent manner (*P *< 0.05). This treatment also increased the number of cells in G0/G1 phase and decreased those in G2/S/M phase.

**Conclusions:**

The hypoxia-mimetic agents, DFO and CoCl_2_, alter umbilical cord-derived hMSCs morphology and inhibit their proliferation through influencing the cell cycle.

## Background

Human mesenchymal stem cells (hMSCs) were first identified by Friedenstein et al. [1] in 1974. As non-hematopoetic, multipotent bone marrow stem cells, hMSCs are more primitive and embryonic-like cells with the potential to differentiate into lineage-committed progenitors and mature cells, such as osteoblasts and fibroblasts [2]. However, bone marrow is not the exclusive source of MSCs; they have been isolated from virtually all post-natal and extra-embryonic tissues, including amniotic membrane, placenta, umbilical cord, and umbilical cord blood [3-5]. In recent years, hMSCs have been commonly used in tissue engineering, cell replacement therapy, gene therapy, and body organ/fluid transplantation.

Oxygen is a potent signaling molecule, affecting the fundamental characteristics of various types of cells. Specifically, reduced oxygen levels or hypoxia influences blood-brain barrier permeability through influencing endothelial cell junctional complexes [6]. Furthermore, hypoxia induced proliferation of hematopoietic bone marrow stem cells [7]. In addition, hypoxia-inducible factor-1 alpha (HIF-1α), a critical transcription factor in the mammalian oxygen-sensing pathway, is activated in response to hypoxia, altering tumor xenograft gene expression, growth, and angiogenesis [8] possibly through membrane type 1 metalloprotease [9]. MSCs exposed to hypoxic conditions exhibit greater colony-forming potential [10], faster and prolonged proliferation [11-13], and greater chemotaxis [9]. Exposure of MSCs to hypoxia also prolonged their differentiation [14].

Hypoxia is often induced by decreasing oxygen concentrations [15,16]. In addition, the hypoxia-mimetic agents, cobalt chloride (CoCl_2_) and desferrioxamine (DFO), an iron chelator, artificially induce hypoxia through blocking the degradation of HIF-1α [17,18]. The effects of hypoxia-mimetic agents are comparable to those resulting from reduced atmospheric oxygen levels [17,19].

Analysis of stem cell proliferation and differentiation is often carried out in 20% O_2_, which is much greater than that found *in vivo*; arterial oxygen concentration is approximately 12% while bone marrow ranges from 1 to 7% [20]. Therefore, the effects of DFO and CoCl_2 _on human umbilical cord-isolated hMSCs were assessed. The ultrastructure of hMSCs in response to hypoxia was analyzed using atomic force microscopy (AFM) and transmission electron microscopy (TEM). In addition, the effects of hypoxia mimetics on hMSCs proliferation were analyzed. Determining the effects of hypoxia on hMSCs morphology and proliferation may contribute to identifying the mechanisms by which hypoxia influences hMSCs chemotaxis and migration as well as characterize their therapeutic application for hypoxia-related disease.

## Results

### Morphological and immunophenotypic characterization of umbilical cord-derived hMSCs

**Figure 1:**
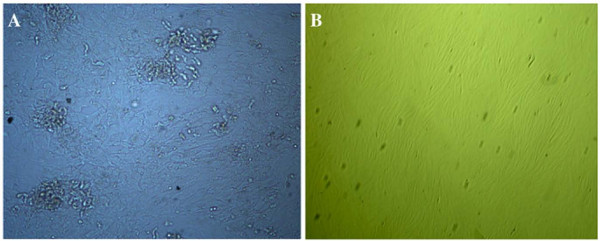
**Morphological changes in hMSCs primary cultures over time**. A) First passage at 5 days (100×). B) Third passage at 3 days (50×).

Both large and occasionally multi-nucleated hMSCs as well as small, spindle-shaped mononucleated cells were present in the primary hMSCs culture (Figure 1A). Following the second passage, the cultures were primarily made up of the small, spindle-shaped cells, which continued to proliferate after numerous passages; the number of large hMSCs diminished (Figure 1B). Individual spindle-shaped cells appeared after 3-4 days in culture, while colonies formed as early as 5 days.

**Figure 2:**
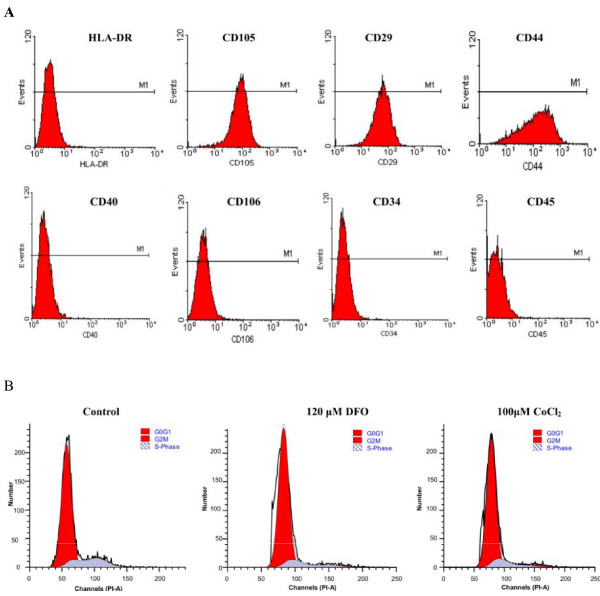
**Histograms representing the immunophenotype and cell cycle distribution as determined by flow cytometry**. The phenotype of hMSCs was determined after cell isolation (A). The cell cycle distribution was determined without (control) or with 120 μM DFO or with 100 μM CoCl_2 _for 48 h (B).

The primary hMSCs expressed CD44 (96.1%), CD29 (98.5%), and CD105 (98.6%) surface antigens whereas expression of the CD106 (2.1%), CD40 (0.8%), CD34 (0.5%), CD45 (0.8%), and HLA-DR (0.7%) surface molecules were below the detection limit (Figure 2A).

### hMSCs differentiation

**Figure 3:**
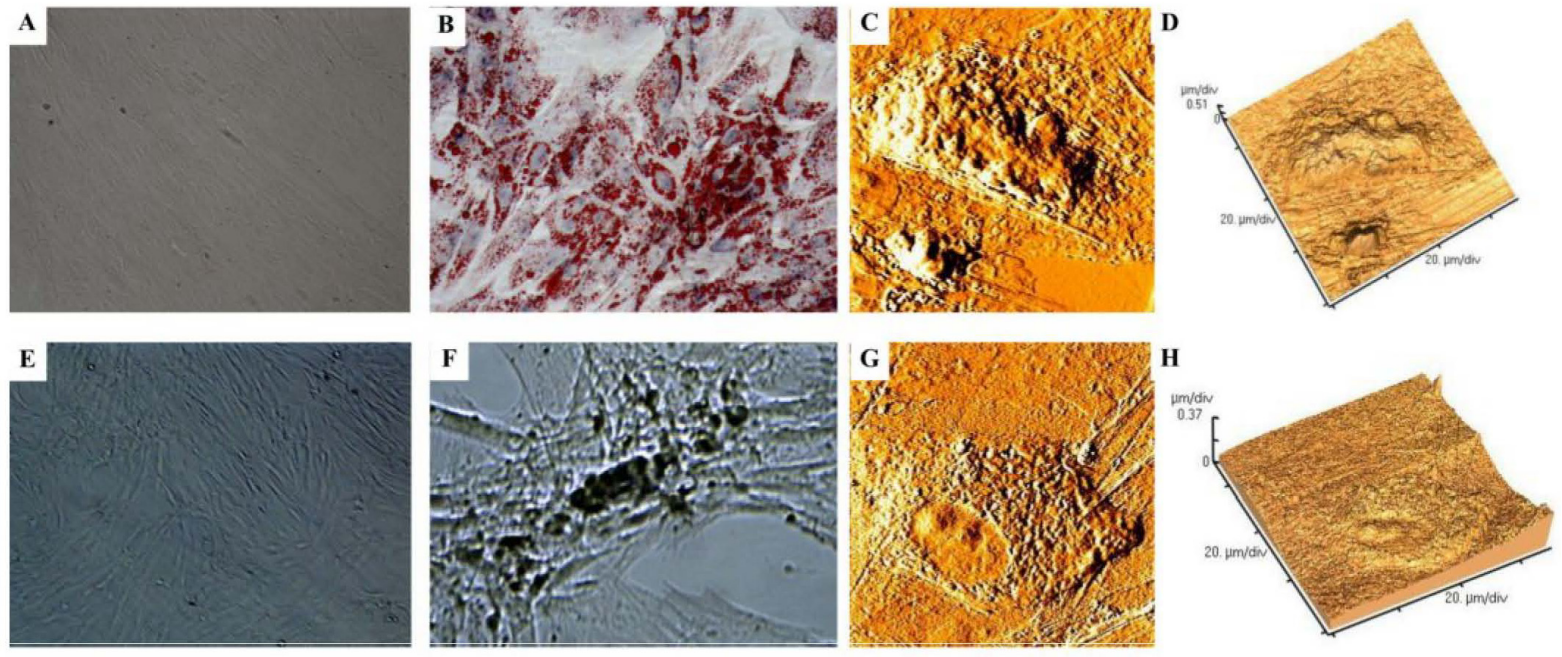
**Adipogenic and osteogenic differentiation of hMSCs**. Adipogenesis was detected by the formation of intracytoplasmic lipid droplets stained with oil red O (A, no induction; B, induced cells; 100×). Osteogenic differentiation was demonstrated by calcium deposition as evidenced von Kossa staining (E, no induction; F, induced cells; 100×). Atomic force microscopy of umbilical cord-derived hMSCs upon adipogenic (C, D) and osteogenic (G, H) differentiation after 21 days in normoxic conditions were shown as error signal images (C,G) and 3-dimension graphs (D,H).

Following adipogenic induction, hMSCs morphology changed from elongated, confluent fibroblastic-like cells (Figure 3A) to oval-shaped cells; a distinct ring of red coarse vacuoles around the cell periphery was observed upon Oil Red O staining after the fourth day, becoming larger and more numerous over time (Figure 3B). Upon osteogenic differentiation, adherent monolayers of spindle-shaped cells (Figure 3E) became multilayered cell clusters surrounded by a matrix-like substance that was visible after von Kossa staining (Figure 3F). In addition, rapid mineralization and nodule formation characterized by the accumulation of overcrowded fibroblast-like cells in direct contact with one another was observed. The cells bordering the nodules were fibroblastic, while those near the centers were polygonal (Figure 3F).

### Effects of DFO and CoCl_2 _on hMSCs proliferation

**Figure 4:**
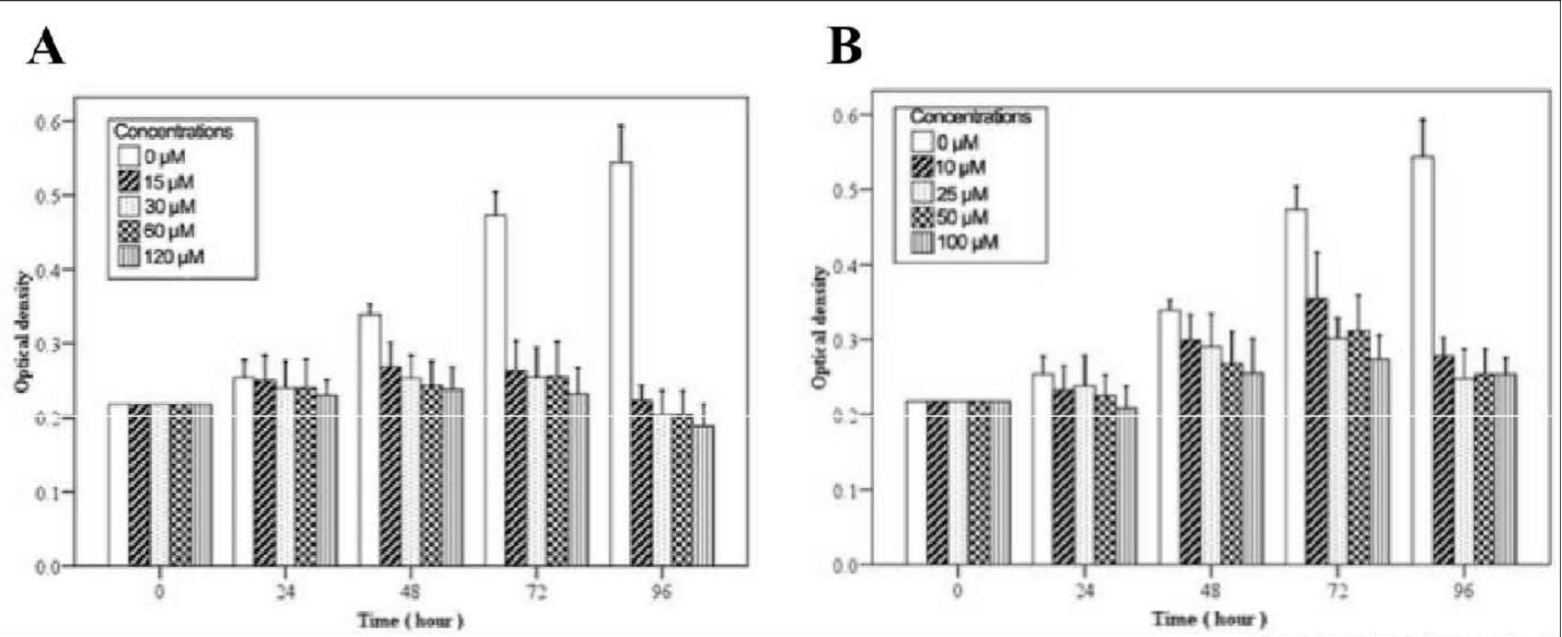
**Reduced hMSCs growth after treatment with hypoxia mimetics**. A) hMSCs were treated with the indicated concentration of A) DFO or B) CoCl_2 _at various time points. Data represent the mean ± SD. *P < 0.05 as compared to the control group.

hMSCs proliferation rates were determined using the MTT assay; logarithmic growth was observed after 48 h. Decreased hMSCs proliferation was detected after DFO (Figure 4A) and CoCl_2 _(Figure 4B) treatment. Specifically, significantly decreased cell growth was observed after treatment with 120 μM DFO and 10 and 100 μM CoCl_2 _as compared to the untreated controls (all *P *< 0.05). After 48 h, the inhibition rates were 20.6, 25.2, 28.0, and 29.8% for cells treated with 15, 30, 60 and 120 μM DFO, respectively (*P *< 0.05). Similarly, the inhibition rates were 11.7, 14.2, 20.9, and 24.6% for hMSCs treated for 48 h with 10, 25, 50, and 100 μM CoCl_2_, respectively (*P *< 0.05).

### Effects of hypoxia mimetics on hMSCs cell cycle

As compared to the control group, the ratio of hMSCs in the G0/G1 phase increased while those in the G2/M/S phase decreased after DFO and CoCl_2 _treatment (Table 1; Figure 2B). Although no significant differences were observed among the various DFO or CoCl_2 _concentrations, an increasing trend was observed in the number of cells in the G0/G1 phase while a decreasing trend was detected in the number of cells in the G2/S/M phase after treatment (Table 1; Figure 2B).

**Table 1:** Effects of DFO and CoCl_2 _of different concentrations on cell cycle distribution of hMSCs detected by flow cytometry with PI staining

		DFO (μM)		CoCl_2 _(μM)	
			
Cell phases	Control	15	120	25	100
G0/G1 (%)	80.03 ± 4.28	82.67 ± 3.25	86.41 ± 3.13	81.96 ± 2.21	85.87 ± 2.93
G2/M (%)	1.52 ± 0.57	1.35 ± 1.29	1.30 ± 1.32	1.37 ± 0.29	1.32 ± 0.74
S (%)	18.45 ± 4.10	15.98 ± 4.21	12.29 ± 1.95	16.67 ± 2.13	12.81 ± 2.87

(n = 3)

### Effects of hypoxia mimetics on hMSCs morphology

**Figure 5:**
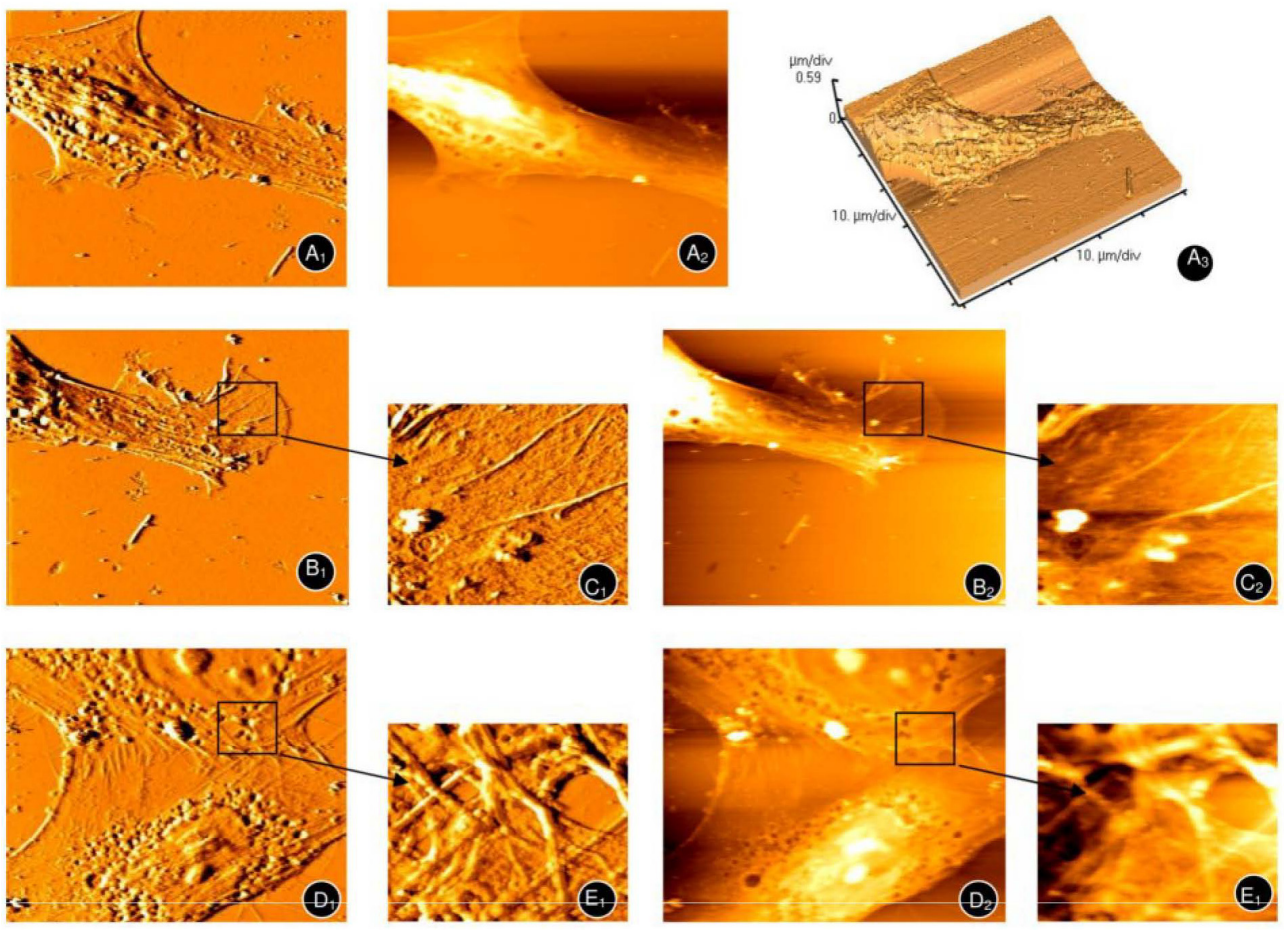
**Atomic force microscopy of hMSCs under normoxic conditions**. A typical long spindle, cytoskeleton (A_1_, A_2_, A_3_), and palpus-like or cicada wing-like pseudopodium (B_1_,B_2_, enlarged areas shown in C_1 _and C_2 _respectively) were observed. The mesh-like cytoskeleton of adjacent hMSCs (D_1_, D_2_) can also been seen (enlarged areas shown in E_1_, E_2_).

After 72 h in normoxic conditions, AFM revealed enlarged, spindle-shaped hMSCs up to 80 μm in length and 30-40 μm wide (Figures 5A1, A2 and 5A2). In addition, a clear cytoskeleton, complex edge as well as synapse-like structure, and fishtail-like or cicada wing-like morphology were observed (Figures 5B1 and 5B2, enlarged areas shown in 5C1 and 5C2). In addition, cells were in close contact with each other and presented a reticular structure, suggesting active cell-to-cell signaling (Figures 5D1 and 5D2, enlarged areas shown in 5E1 and 5E2).

hMSCs were exposed to factors inducing adipogenic and osteogenic differentiation for 21 days in normoxic conditions after which the morphology was assessed. The nuclei of adipogenic-induced hMSCs moved to peripheral area while the cell membranes became more granular, which was suggestive of the S phase of the cell cylce (Figures 3C and 3D). Osteogenic-induced hMSCs appeared more elevated, square and rigid than undifferentiated cells (Figures 3G and 3H).

**Figure 6:**
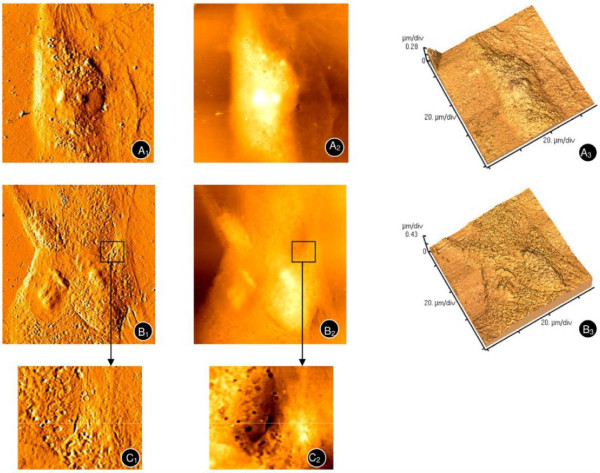
**Atomic force microscopy of hMSCs upon treatment with hypoxia mimetics**. hMSCs were treated with 120 μM DFO (A_1_, A_2_, A_3_) and 100 μM CoCl_2 _(B_1_, B_2_, B_3_) for 4 days. Enlarged areas of B_1 _and B_2 _are shown in C_1 _and C_2 _respectively.

Altered hMSCs morphology was observed after treatment with DFO (Figures 6A1, A2 and 6A3) and CoCl_2 _(Figures 6B1, B2, and 6B3, enlarged areas of 6B1 and 6B2 shown in 6C1 and 6C2, respectively). Specifically, the spindle-like cells elongated up to nearly 100 μm. In addition, cell-to-cell contacts diminished, and gaps appeared in between adjacent cells.

**Figure 7:**
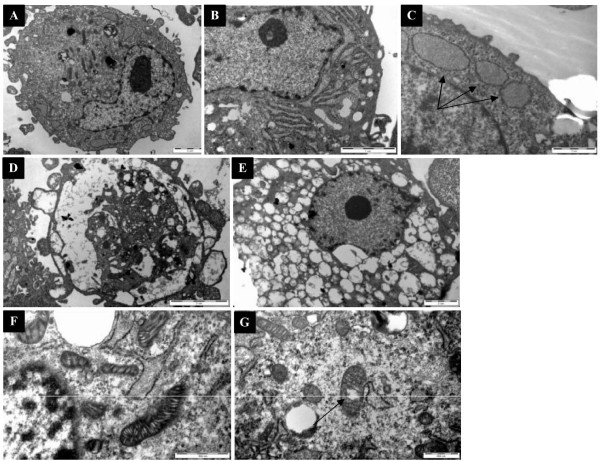
**Transmission electron microscopy of hMSCs**. A) Complete view of untreated (8900×) or D and E) DFO-treated hMSCs after 3 days (8900×). (B and C) The endoplasmic reticulum of untreated hMSCs (24000×). F) The mitochondria of untreated (30000×) and G) DFO-treated hMSCs (30000×).

TEM analysis of control hMSCs revealed round cells with surface protrusions and microvilli (Figure 7A). The nuclei were irregularly shaped with ≥ one nucleoli, containing a large amount of euchromatin and a smaller amount of heterochromatin. With the exception of a small amount of rough endoplasmic reticulum (Figures 7B and 7C), ribosomes, and mitochondria (Figure 7F) with clear and continuous cristae, relatively few organelles were found in the cytoplasm, suggesting that they had remained undifferentiated. After treatment with DFO, more surface protrusions and microvilli, as well as a large number of intracellular vacuole-like structures were observed (Figures 7D and 7E). In addition, DFO treatment resulted in shrinkage or partial disintegration of the nuclear membrane, as well as chromatin condensation. Furthermore, an expanded rough endoplasmic reticulum, which was filled with a low density matrix, was detected. Although the number and volume of mitochondria were not significantly different from the control groups, mitochondria with an expanded ridge and containing a high density matrix were observed in DFO-treated hMSCs (Figure 7G). Significant changes in other organelles were not observed.

## Discussion

Human umbilical cords represent a promising source of hMSCs, which can be conveniently isolated and induce low immunogenicity. In addition, as compared to other stem cell sources these present relatively few ethical issues. The hMSCs isolated from umbilical cords were positive for CD44, CD29 and CD105, and negative for CD106, CD40, CD34, CD45 and HLA-DR surface antigens, confirming their identity as MSCs [21]. In addition, the isolated hMSCs were able to differentiate into adipogenic and osteogenic cells under specific culture conditions.

The intracellular cytoskeleton responds to external mechanical stimuli [22]. Tapping mode AFM was used to observe the surface structures. The position of the adipogenic cell nuclei and their granular quality of the membranes is suggestive of early S phase of the cell cycle. Osteogenic-induced hMSCs appeared larger, squarer and more rigid as compared to undifferentiated cells, which is consistent with that reported by Danti et al. [23]. In addition, they were easily distinguishable from each other as the differentiation process prevented their proliferation, which also correlates with Danti et al [23].

Differentiation of MSCs is also influenced by hypoxia. Qu et al. [24] reported that DFO increased the osteoblastic differentiation of bone morphogenetic protein-2-treated MSCs. Valorani et al. [25] and Ren et al. [12] observed that hypoxia (2 and 8%, respectively) increased the adipogenic differentiation potential of MSCs. These studies suggest that pre-culturing MSCs under hypoxic conditions prior to transplantation may enhance their efficacy.

As evidenced by clinical trials in hypoxic-ischemic diseases, MSCs-based therapy has potential value in tissue replacement and regeneration [26-28]. The effects of *in vivo *oxygen concentrations, which are much lower than common experimental conditions, on hMSCs were largely unknown. Therefore, hMSCs proliferation and morphology in response to hypoxia induced by DFO and CoCl_2 _was assessed. After treatment with DFO and CoCl_2_, hMSCs were more elongated, and gaps appeared between adjacent cells. Thus, DFO and CoCl_2 _may inhibit hMSCs growth by weakening cell-to-cell signaling. The reduction in cell-cell junctions may also mediate hMSCs migration induced by hypoxia (3% oxygen) [29]. Further studies are necessary to determine the effects of DFO and CoCl_2 _treatment on the signaling pathways that govern hMSCs migration.

The influence of hypoxia on hMSCs ultrastructure was also explored by TEM. After treatment with DFO, hMSCs contained a large number of unidentifiable vacuoles that are early markers of apoptosis, which is consistent with Ren et al. [12] using 8% oxygen. In addition, the observed shrinkage, disintegration, and dissolution of the nucleus, along with chromatin condensation indicated early apoptosis. Consistent with the signs of apoptosis, we found that hMSCs proliferation decreased in a dose-dependent manner with increasing concentrations of CoCl_2 _and DFO. However, discrepant effects of physical hypoxia and hypoxia mimetics on MSCs proliferation indicate differences. For example, Ren et al. [12] reported that low oxygen levels (8%) promoted MSCs proliferation, whereas DFO (120 μM) and CoCl_2 _(100 μM) inhibited their growth. Lavrentieva et al. [30] reported that 1.5-5% oxygen levels increased the proliferative capacity of hMSCs. Whereas Qu et al. [24] found that DFO, ranging from 0 to 100 μM, inhibited cell growth in a dose-dependent manner. HIF-1α levels may differ under physically-induced hypoxia as opposed to CoCl_2_-induced hypoxia [12,16] which may account for these differences; however, further analysis is required.

The effects of CoCl_2 _and DFO on hMSCs proliferation may be mediated by cell cycle changes. A larger percentage of DFO- and CoCl_2_-treated cells in the G0/G1 phase was observed, while the ratio of those in the G2/M/S phase decreased. Similar results were reported by Holzwarth et al. [31], who reported hMSCs accumulation in the G1 phase at 1% oxygen. Further studies are necessary to determine if the cell cycle effects of hypoxia mimetics can be recapitulated under low oxygen conditions.

There are several study limitations that warrant discussion. Firstly, the present study analyzed the effects of hypoxia mimics on hMSCs; however, the effects of physical hypoxia were not assessed. Further studies will be carried out to compare the effects of physical hypoxia and hypoxia mimetics on hMSCs morphology and growth. In addition, the effects of DFO and CoCl_2 _on hMSC HIF-1α levels were not analyzed. However, previous studies using the same concentrations of DFO and CoCl_2 _used in the present study have reported upregulation of HIF-1α expression [12,32]. Furthermore, the present study did not analyze the influence of DFO and CoCl_2 _on MSCs differentiation. Although the effects of these agents on MSCs morphology is suggestive of greater self-renewal capacity as hMSCs broaden and flatten with differentiation [33], and differentiation was associated with changes in nuclear morphology [34,35], determining their effects on MSCs differentiation will be the focus of future studies. Finally, the effects of hypoxia-induced morphological changes on MSCs function (e.g., cell migration, homing or immune regulatory effect) was not explored in the present study, but will be analyzed in future studies. Although the present study has its limitations, determining the ways in which MSCs respond to environments with lower than atmospheric oxygen concentrations, such as the blood, bone marrow, and cartilage, is crucial for their successful use regenerative medicine. The present study advances our understanding of the influences of hypoxia on MSCs morphology and proliferation.

## Conclusions

We isolated and cultured hMSCs from umbilical cords. The proliferation of hMSCs was inhibited by DFO and CoCl_2_. The cell surface and ultrastructure was viewed with atomic force microscopy and transmission electron microscopy after DFO- and CoCl_2_-induced hypoxia, which is the first time this has been reported. These data will provide a better understanding of the potential therapeutic applications of hMSCs in hypoxic-ischemic disease.

## Methods

### Isolation and culture of hMSCs from umbilical cord and treatment with hypoxia-mimetic agents

hMSCs were obtained from the umbilical cords of healthy full-term infants born by cesarean section. Tissues were washed with phosphate buffered saline (PBS), cut into approximately 1 cm^3 ^pieces, and digested with 0.2% type II collagenase (Gibco, USA) at 37°C for 5-6 h. After filtration and centrifugation, the cells were incubated in DMEM/F12 medium supplemented with 10% fetal bovine serum (FBS, Gibco, USA), 100 U/mL penicillin and 100 U/mL streptomycin. Cells were inoculated at density of 1 × 10^6 ^cells/mL in 25 cm^2 ^culture flasks (Corning, NY) and maintained at 37°C in a humidified atmosphere containing 5% CO_2_. After 4-5 days, non-adherent cells were removed. The culture medium was replaced every 3 days, and adherent cells were cultured until they reached 80-90% confluence. Cells at passage 3-7 were used to analyze the effects of hypoxia-mimetic agents. Informed consent for the use of the umbilical cords was obtained from each mother prior to surgery. The study was approved by The Ethics Committee of the First Affiliated Hospital, Jinan University, Guangzhou, China.

DFO and CoCl_2 _were dissolved in ultrapure water and applied to the hMSCs cultures at concentrations of 10, 25, 50, and 100 μM for CoCl_2 _and 15, 30, 60, and 120 μM for DFO. Control groups consisted of cells grown in the absence of DFO and CoCl_2_.

### Immunophenotype determination and cell cycle analysis

hMSCs were incubated with either phycoerythrin or FITC-conjugated antibodies specific for CD29, CD44, CD90, CD105, CD106, CD40, CD34, and CD45 (BD Biosciences, San Jose, CA). Mouse isotype control antibodies served as negative controls. Cells were stained with a single label and analyzed by flow cytometry using a BD FACSCalibur.

Cell cycle was analyzed through propidium iodide (PI)-staining. hMSCs (10^6 ^cells) in the third passage were fixed, permeabilized in 70% cold ethanol at 4°C for 24 h, and treated with 200 μg/mL RNase A for 30 min at 37°C. hMSCs were then incubated in 40 μg/mL PI for 5 min at room temperature, and placed on ice until analysis by flow cytometry.

### Analysis of hMSCs differentiation

To evaluate hMSCs properties, adherent cells (in the third passage, at 80-90% confluence) were subjected to adipogenic and osteogenic differentiation *in vitro*, according to established protocols [36,37]. For adipogenic differentiation, cells were incubated in DMEM/F12 media supplemented with 10% FBS, 100 U/mL penicillin, 100 U/mL streptomycin, 5 μg/mL insulin, 50 mM indomethacin, 1 μM dexamethasone, and 0.5 mM 3-isobutyl-1-methylxanthine. Adipogenic differentiation was confirmed on the 21st day by observing the intracellular accumulation of lipid-rich vacuoles stained with oil red O. For osteogenic differentiation, cells were incubated in DMEM/F12 media supplemented with 10% FBS, 100 U/mL penicillin, 100 U/mL streptomycin, 10 mM b-glycerol phosphate, 100 nM dexamethasone, and 50 μM ascorbate 2-phosphate for 21 days. Osteogenic differentiation was confirmed by the accumulation of mineralized calcium phosphate and assessed using the von Kossa staining method, which entails incubating the cells in 1% silver nitrate for 60 min under ultraviolet light followed by 3% sodium thiosulfate for 5 min at room temperature.

### MTT proliferation assay

After treatment, cell growth media was replaced with 200 μL MTT solution (5 g/L in PBS) per well for 4 h after which it was replaced with 150 μL dimethyl sulphoxide. Quantification was then carried out using a microplate reader at 570 nm; a 630 nm filter was used as a reference.

### Atomic force microscopy

hMSCs were cultured on glass coverslips in growth media alone (control group) or containing 120 μM DFO or 100 μM CoCl_2_. After the cells reached 70-80% confluence at 72 h, they were gently washed with PBS, fixed with 4% paraformaldehyde, washed with demineralized water, and air dried. Tapping-mode atomic force microscopy analysis (Autoprobe CP research, Thermomicroscopes, California, USA) was performed with silicon nitride tips (spring constant, 0.9 N/m) using a Park Scientific Instruments commercial instrument (MPP-31123). The scanning speed was 0.4-1HZ. The IP2.1 software (Thermomicroscopes Proscan Image Processing Software Version 2.1, California, USA) applied smoothing analysis to the resulting images.

### Transmission electron microscopy

TEM was used to assess the morphological characteristics of hMSCs. hMSCs cultured for 72 h in media containing 120 μM DFO were immediately fixed at 4°C with 2.5% glutaraldehyde, post-fixed in osmium, and routinely processed for TEM assessment (PHILIP5 TECNAI 10, HOLLAND). Cells cultured in complete medium alone were used as a control group.

### Statistical analysis

Statistical analyses were performed using SPSS 15.0 statistics software (SPSS Inc, Chicago, IL, USA). Data were expressed as mean ± standard deviation (SD). One-way ANOVA test with a post-hoc Bonferoni comparison was performed to compare the data among concentrations. Furthermore, a repeated ANOVA test for repeated cell growth was performed to compare the data among concentrations. Finally, a generalized linear model analysis was applied to show changes in data over time. All *P*-values < 0.05 were considered significant.

## Authors' contributions

HLZ participated in the design of the study and drafted the manuscript .QZ carried out atomic force microscopy and transmission electron microscopy, and drafted the manuscript .YLQ carried out the isolation and culture of hMSCs and their immunophenotype characterization. QQB carried out the analysis of hMSC differentiation. XAH carried out the MTT proliferation analysis. HTJ performed the statistical analysis. HWL participated in the design of the study and edited the manuscript. All authors read and approved the final manuscript.
